# Supplementary material for: Actin crosslinker competition and sorting drive emergent GUV size-dependent actin network architecture
Source: Commun Biol. 2021 Sep 28;4:1136. doi: 10.1038/s42003-021-02653-6 (PMC8478941; doi:10.1038/s42003-021-02653-6)
Supplement: Supplementary file 2 — Supplementary Information [file 42003_2021_2653_MOESM2_ESM.pdf]

Supplemental Information

Actin crosslinker competition and sorting drive emergent GUV size-dependent actin network architecture

Yashar Bashirzadeh[1], Steven A. Redford[2,3], Chatipat Lorpaiboon[4], Alessandro Groaz[1,*], Hossein Moghimianavval[1], Thomas Litschel[5], Petra Schwille[5], Glen M. Hocky[6], Aaron R. Dinner[2,4], Allen P. Liu[1,7,8,9]

[1] Department of Mechanical Engineering, University of Michigan, Ann Arbor, Michigan, 48109, USA

[2] James Franck Institute, University of Chicago, Chicago, Illinois, 60637, USA

[3] The graduate program in Biophysical Sciences, University of Chicago, Chicago, Illinois, 60637, USA

[4] Department of Chemistry, University of Chicago, Chicago, Illinois, 60637, USA

[5] Department of Cellular and Molecular Biophysics, Max Planck Institute of Biochemistry, 82152, Martinsried, Germany

[6] Department of Chemistry, New York University, New York, NY, 10016, USA

[7] Department of Biomedical Engineering, University of Michigan, Ann Arbor, Michigan, 48109, USA

[8] Department of Biophysics, University of Michigan, Ann Arbor, Michigan, 48109, USA

[9] Cellular and Molecular Biology Program, University of Michigan, Ann Arbor, Michigan, 48109, USA

* Current address: Department of Neuroscience, Baylor College of Medicine, Houston, Texas, 77030, USA

Corresponding authors:

A.R.D.: dinner@uchicago.edu; 929 E 57th Street, University of Chicago, Chicago, Illinois 60637. Tel: +1 773-702-2330.

A.P.L: allenliu@umich.edu; 2350 Hayward Street, University of Michigan, Ann Arbor, Michigan 48109. Tel: +1 734-764-7719.

Supplementary Methods

Image Processing

Z-stack confocal images of actin-labelled vesicles were mostly analyzed using ImageJ/Fiji[1, 2], complemented with the plugin Squassh[3] from the MOSAIC ToolSuite update site. Encapsulated actin structures were characterized using ImageJ/Fiji in combination with SOAX[4, 5]. Specifically, a skeleton model from the z-stack confocal images was derived as a combination of ImageJ scripts, each one performing a processing step on images. After making the images conform to its input specifications, the software SOAX was launched and controlled through Fiji to determine a model of the filaments in batch mode.

For the cases where GUVs slightly moved during imaging or a drift between z-planes of either encapsulated actin networks or GUVs occurred, images were first aligned by translation using ImageJ plugin StackReg[6]. The stacks were filtered and the bundles were segmented from them using the Squassh algorithm as implemented in Fiji[3]. The options for the detection of actin structures with Squassh were as follow: background noise elimination with a window of 1 µm size ; regularization factor: 0.075; elimination of background intensity with threshold determined by the Triangle method; Elimination of segmented regions with linear size smaller than 1 µm; automatic estimation of local intensity; Poisson noise model; soft mask for final segmentation. For single GUVs, the image was first cropped in Fiji to include actin bundle-encapsulating GUV of interest prior to image preprocessing (segmentation). Brightness and contrast of all preprocessed images were then manually intensified using Fiji for optimal skeletonization by SOAX program.

A model for actin network on the segmented bundles in each region of interest was determined via SOAX source code[5]. The program implements a Stretching Open Active Contours method to initiate and generate centerlines, so-called snakes, from each bundle in the population, which is used for quantitative measurements of actin bundles. The SOAX parameter settings to extract the snakes were as follow: Intensity Scaling: 0 (automatic); Gaussian Standard Deviation: 1.2 pixels; Ridge Threshold (Tau): 0.01; Minimum Foreground: 2250; Maximum Foreground: 65535; Snake Point Spacing: 2 pixels; Minimum Snake Length: 17 pixels; Maximum Iterations: 10000; Change Threshold: 0.1 pixels; Check Period: 100; Iterations per press: 100; Alpha: 0.003; Beta: 0.5; Gamma: 2; External Factor: 1; Stretch Factor: 0.3; Number of Background Radial Sectors: 8; Radial Near: 1 pixel; Radial Far: 2 pixels; Background Z/XY Ratio: 3; Delta (snake

points): 1; Overlap Threshold: 1 pixel; Grouping Distance Threshold: 2 pixels; Grouping Delta (snake points): 2; Minimum Angle for SOAC Linking: 1.7453 radians; Init z window: marked. Damp z window: unmarked. SOAX generates a file for converged snakes consisting of snake coordinates (actin bundle coordinates). Delete Snake Mode tool of SOAX was used to manually remove converged snakes due to the presence of artefacts or accumulated unbundled fluorescent actin in processed actin images. The SOAX-generated data containing snake coordinates was then saved as a text file. For the cases where the joins between bundles are part of the characterization of actin structures, snakes were cut at junctions and joins formed by SOAX. The coordinates of actin bundles and joints were than save as a text file.

We then developed MATLAB scripts to read the text files, scale voxels to modify image volumes as isotropic, convert pixels to physical units , reconstruct the text as a Chimera marker file in .cmm format for 3D visualization from all directions via UCSF Chimera[7] version 1.14, and quantify geometrical features of encapsulate actin bundles.

Supplementary Figures

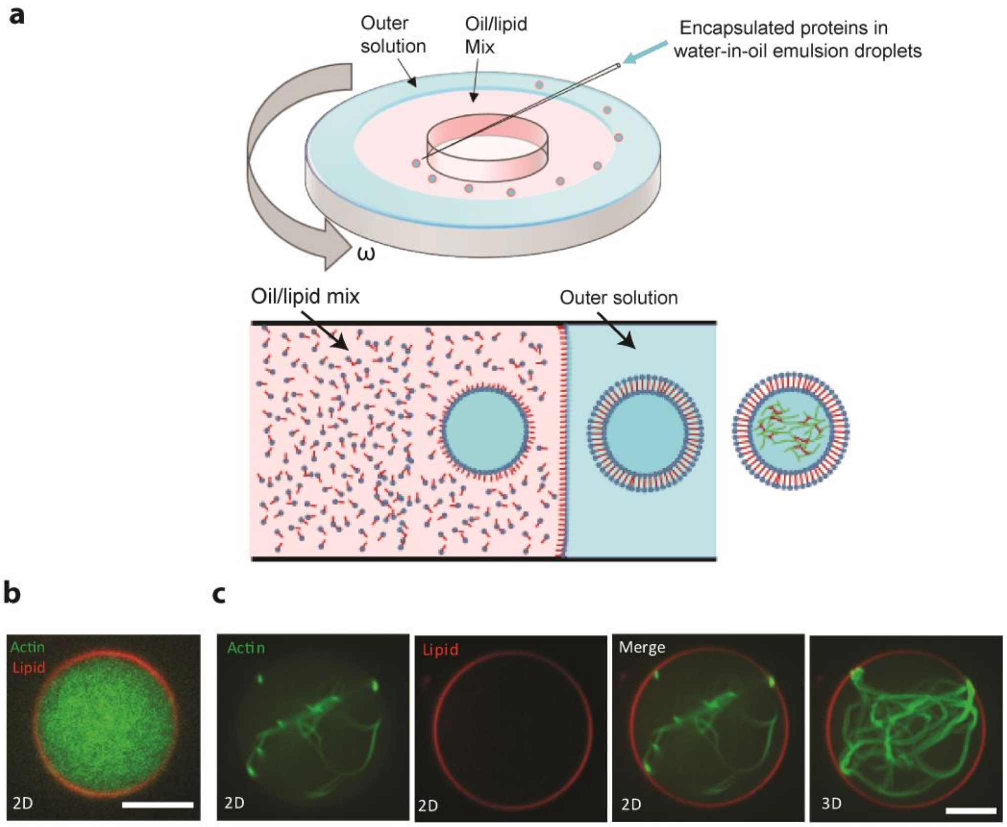

Supplementary Figure 1. Generation of GUVs with encapsulated actin. a, Schematic of the setup for generation of GUVs. **b,** A representative merged (actin:green, lipid:red) confocal image of encapsulated actin in the absence of crosslinkers. Actin, 5 μM, (10% ATTO 488 actin). **c,** Representative confocal fluorescence images from an α actinin-crosslinked actin encapsulated into a large GUV. Actin, green (first from left); membrane, red (second from left); merged image (third from left). 3D-reconstructed image from an image stack, right. Actin, 5 μM (10% ATTO 488 actin). α-actinin, 0.5 μM. Scale bar, 10 μm.

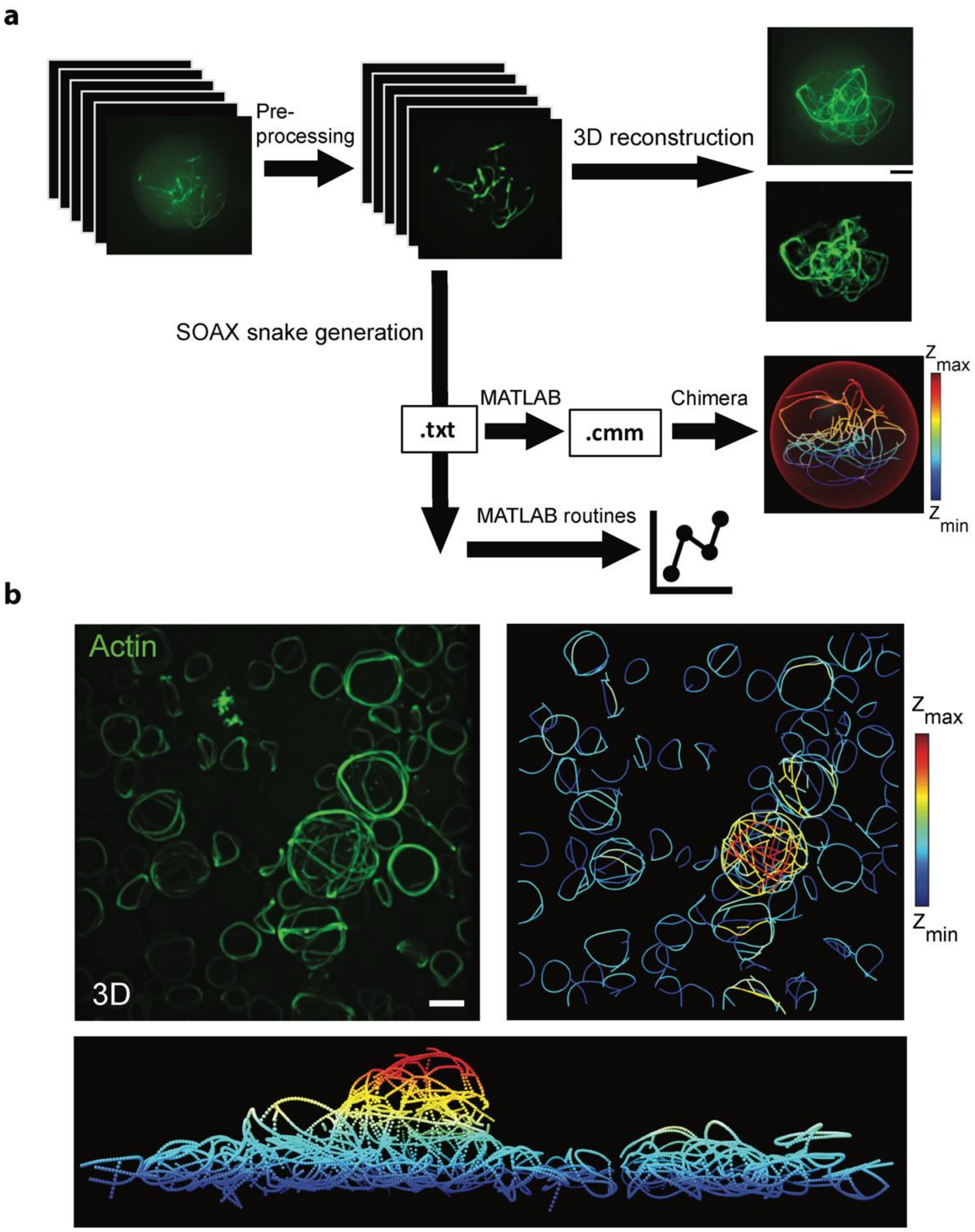

Supplementary Figure 2. Image processing and skeletonization of actin bundles. a, Flow chart depiction of the steps taken to reconstruct 3D actin images, skeletonize bundles via SOAX software, and MATLAB routines for illustration and characterization of encapsulated actin networks. **b,** An example illustrating 3D (left), skeletonized (right), and a skeletonized side view (bottom) image of a population of α actinin-fascin-actin (green) networks encapsulated into GUVs with a wide range of sizes. α -actinin/actin, 0.2 (M/M). Fascin/actin, 0.1 (M/M).

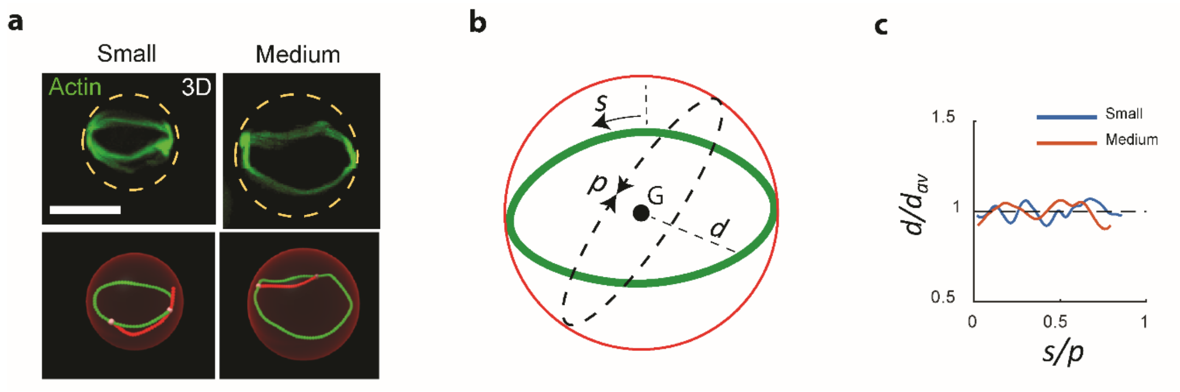

Supplementary Figure 3. Characterization of actin rings. a, Representative 3D actin images (top) and corresponding skeletonized images (bottom) of actin rings in small and medium GUVs. **b-c,** Slight deviation of an actin ring from a circular geometry due to network formation. Scale bar, 10 µm. **b,** Geometrical parameters for characterization of actin rings. d is the distance between ring center of mass and ring points along the ring in skeletonized structures. d_{avg} is the mean value of d (i.e. average ring distance from its center of mass, G). s is arc length along the ring from a starting point on the ring in skeletonized structures. p is the GUV circumference. **c,** Oscillation over $d/d_{avg} = 1$ depicts the deviation of both rings in **(a)** (skeletonized images, green) from the locus of a point defining a circle equidistant from ring center.

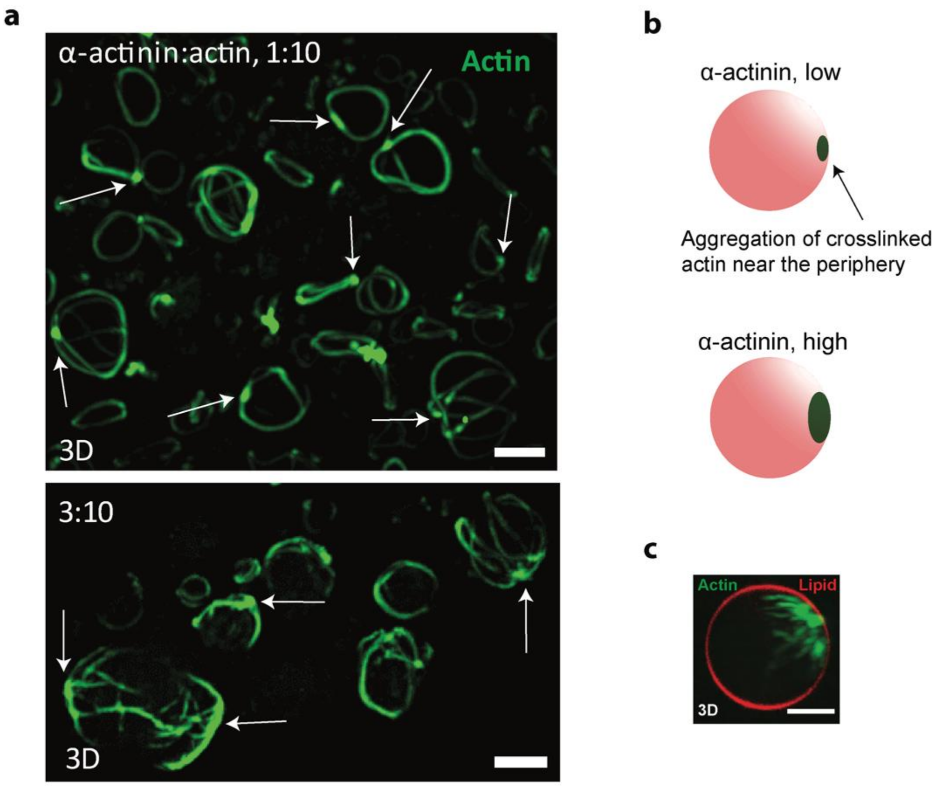

Supplementary Figure 4. a, Representative 3D reconstructed image from confocal fluorescence images of 5 μM actin with α-actinin at α-actinin to actin ratio of 0.1 (top) and 0.3 (bottom) [M/M]. Crosslinked actin forms aster-like structures emanating from actin aggregates (arrows). **b**, Schematic representation of actin aggregation (green) to a focal point at the GUV periphery (pink) via cross-linking activity of α-actinin. Higher α-actinin concentration (right) leads to the formation of a larger and highly localized cluster of actin. **c,** Representative 3D reconstructed image from confocal stack of fluorescence images of an encapsulated α-actinin/actin (3:10 [M/M]) network (rotated view of the same GUV shown in Fig. 1e).

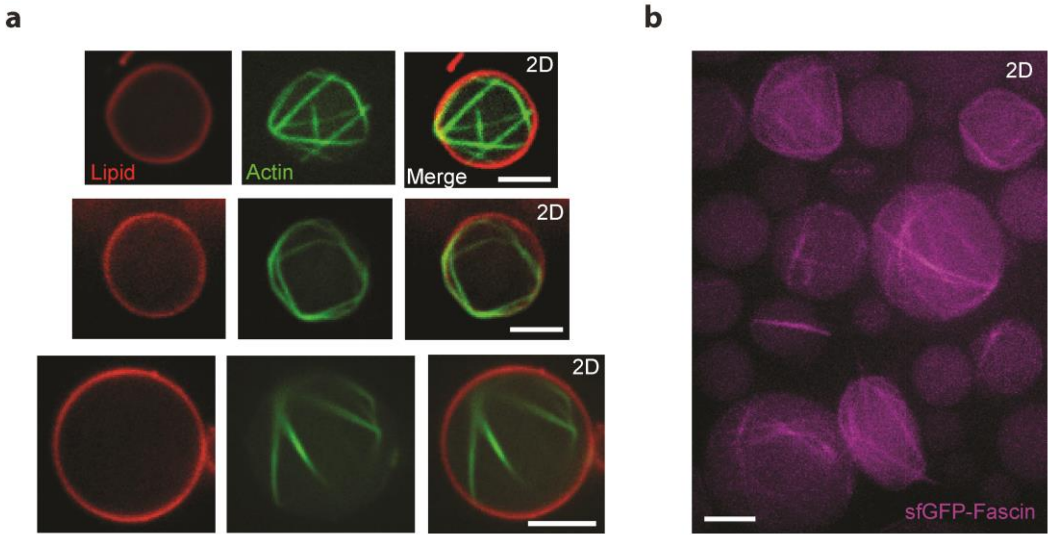

Supplementary Figure 5. The formation of rigid bundle structures by fascin. a, Lipid (left), actin (middle), and merged (right) images of fascin-bundled actin in GUVs with different sizes. Fascin/actin, 0.1 (M/M). Actin, Scale bars, 10 µm. **b,** Confocal fluorescence image of sfGFP-fascin in fascin-actin bundles encapsulated in GUVs. Fascin/actin (including 50 mol% sfGFP-Fascin), 0.1 (M/M). Scale bars, 10 µm.

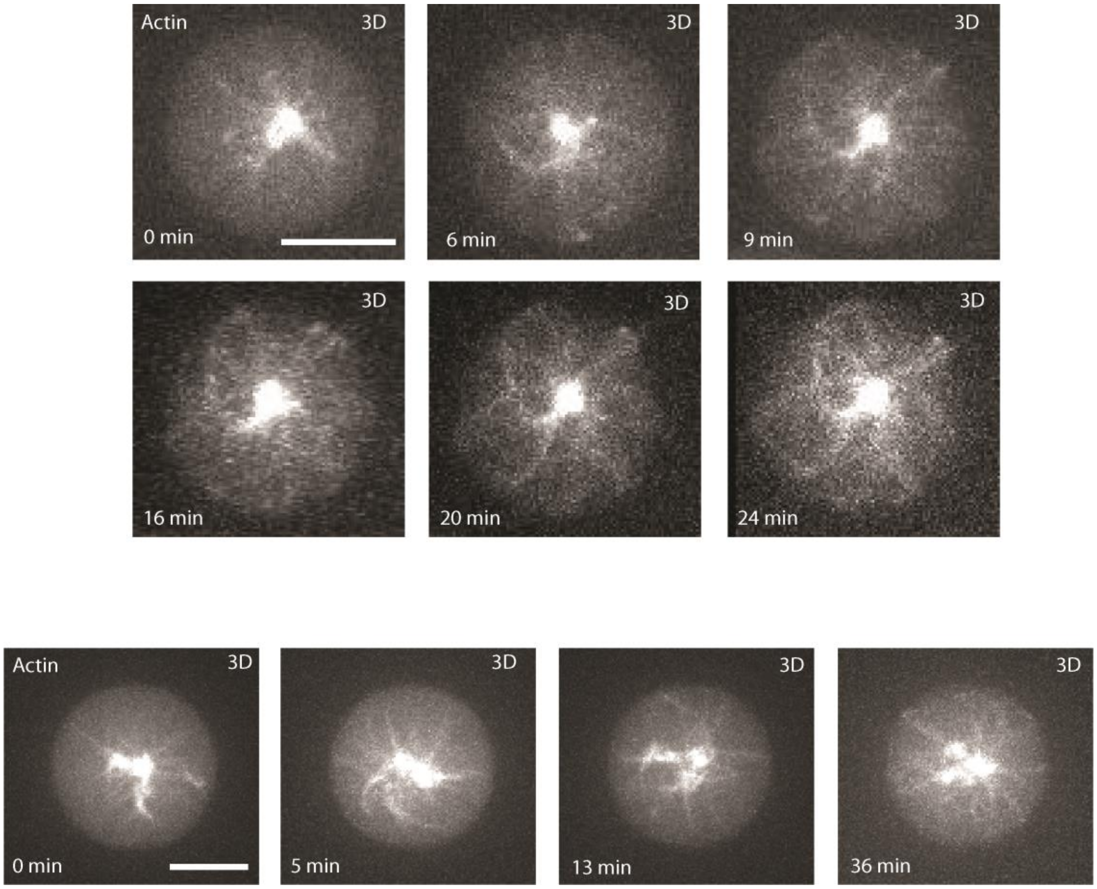

Supplementary Figure 6. Time evolution of bundling activity in central asters. α -actinin/actin, 0.3 (M/M). Fascin/actin, 0.1 (M/M), Scale bar, 10 μm.

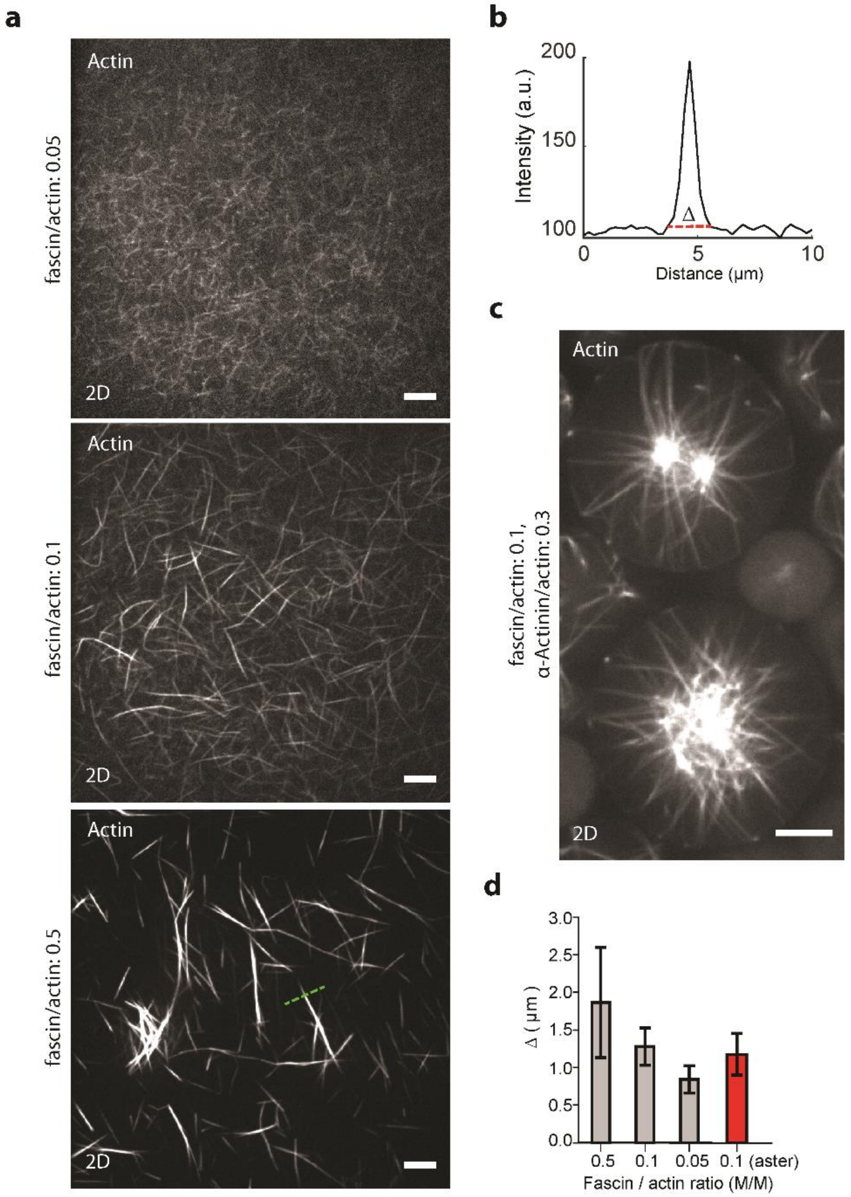

Supplementary Figure 7. a, Representative fluorescence confocal images of actin bundles stabilized with actin stain 488 phalloidin in bulk at molar ratios of fascin indicated. Actin, 5 µM. Scale bar, 10 µm. **b,** Actin fluorescence intensity along the length of green dashed line drawn across an actin bundle in (a). Δ is the maximum distance over which actin bundle intensity is detected along the dashed line. **c,** Representative fluorescence confocal image of encapsulated central asters in the presence of fascin and α-actinin at molar ratios indicated. Actin, 5 µM. Scale bar, 10 µm. **d,** Mean value of Δ obtained for fascin-actin bundles in bulk at indicated molar ratios of fascin (grey bars) and actin bundle arms in encapsulated central actin asters (red bar) at indicated molar ratio of fascin and α actinin. Error bars indicate standard deviation. 20 actin bundles in bulk

were analyzed per condition. In the case of central asters, 20 actin bundles in 10 GUVs were analyzed.

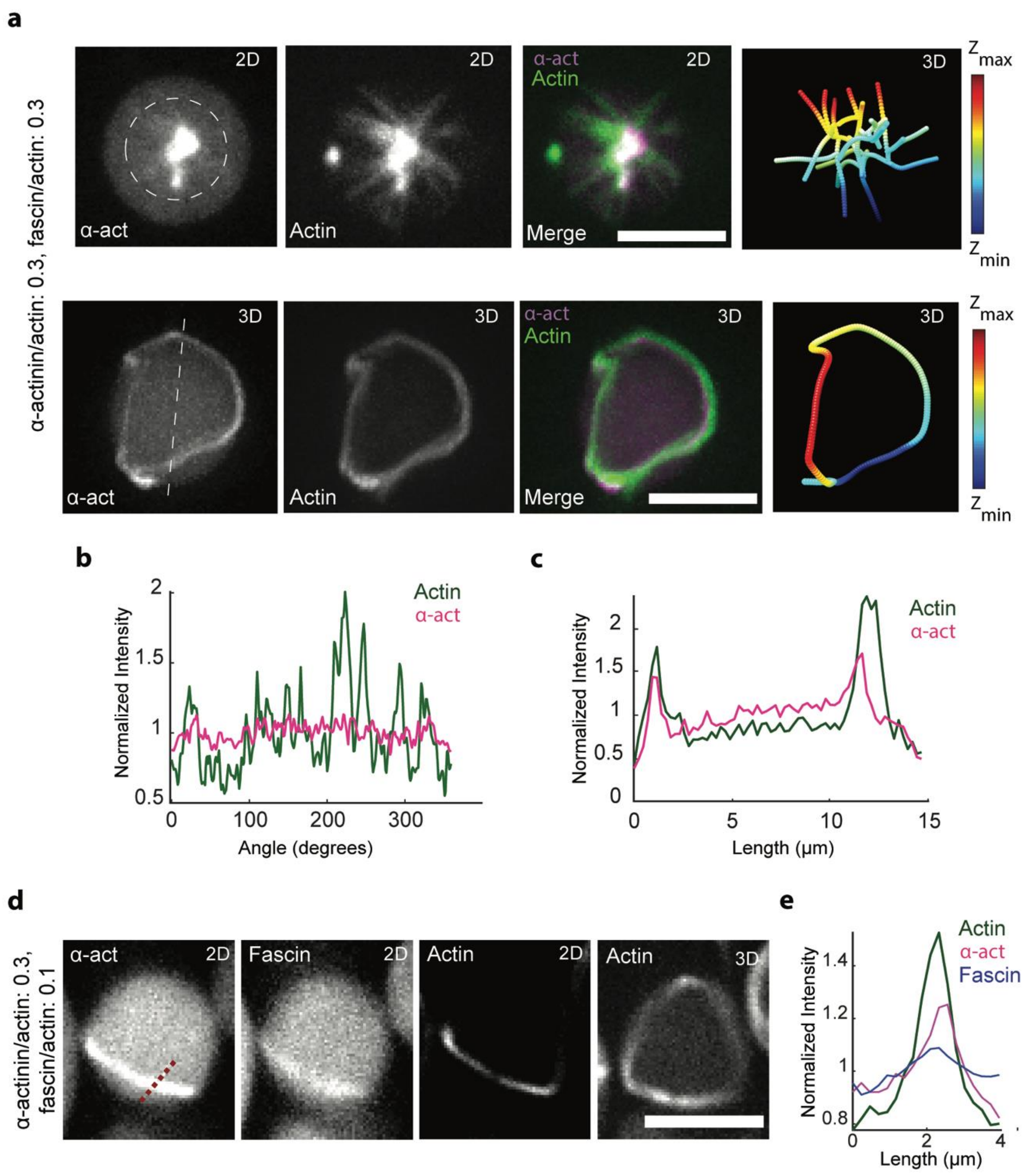

Supplementary Figure 8. The sorting of crosslinkers depends on network architecture. a, Representative 3D reconstructed confocal fluorescence images of α-actinin, actin, merged, and skeletonized construct of an encapsulated star-like actin network (top) and actin ring (bottom) from left to right respectively. α-actinin, 1.5 μM (top), Fascin, 1.5 μM. Actin, 5 μM. Scale bar, 10 μm. **b,** Fluorescence intensity of α-actinin and actin along the circle drawn around star-like structure outside the actin aggregate in (a). **c,** Fluorescence intensity of α-actinin and actin along the dashed line drawn across the actin ring in (a). **d,** Representative confocal fluorescence images of α-actinin,

fascin, and actin in an encapsulated actin ring. α-actinin, 1.5 μM. Fascin, 0.5 μM. Actin, 5 μM. Scale bar, 10 μm. **e,** Fluorescence intensity of fascin, α-actinin, and actin along the red dashed line in (d).

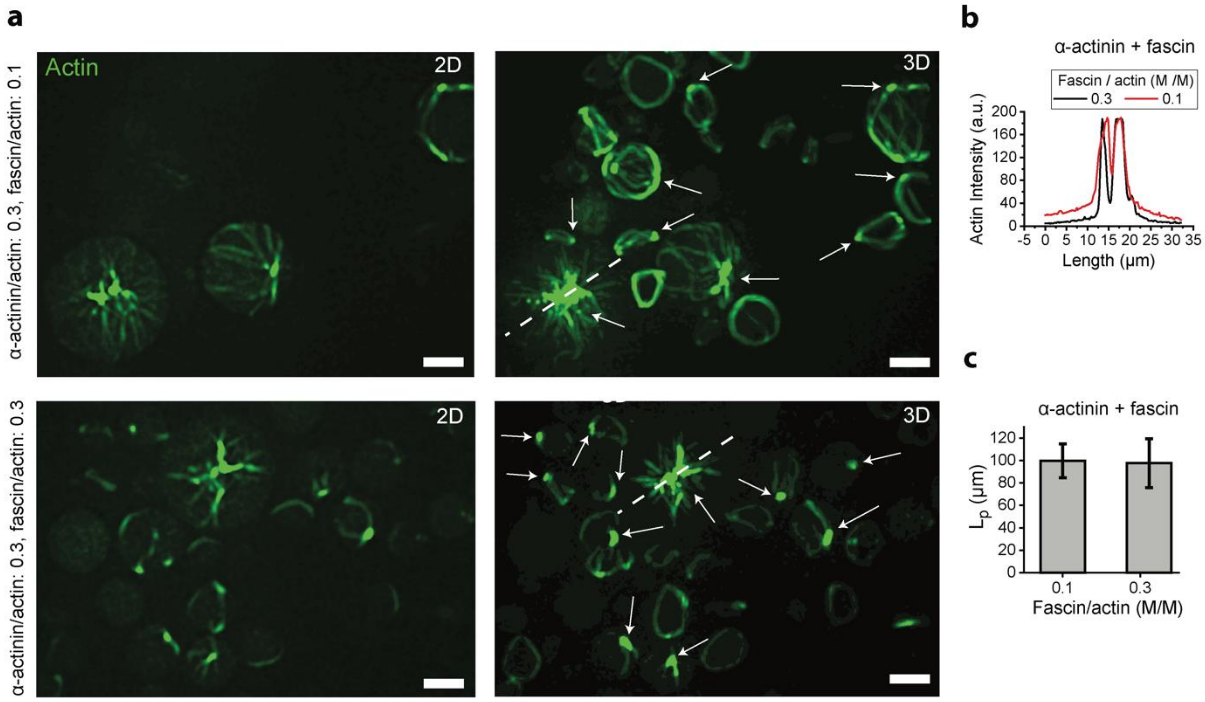

Supplementary Figure 9. At high-α-actinin concentrations, increasing fascin concentration did not change aggregation density nor persistence length of bundles from central asters. a, Representative 2D (left) confocal fluorescence and 3D reconstructed images of actin networks (right). Actin aggregates are indicated by arrows. Fascin, 0.5 µM (top), 1.5 µM (bottom). α-actinin, 1.5 µM. Actin, 5 µM. Scale bar, 10 µm. **b,** Actin fluorescence intensity along the dashed lines drawn across the two GUVs in **(a)**. **c,** Persistence length of actin bundles at two fascin/actin ratios indicated. α-actinin/actin, 0.3 [M/M]). $N_{bundles}$ = [26, 16] in order of x-axis categories; 3 GUVs per category. Error bars indicate standard error of the mean.

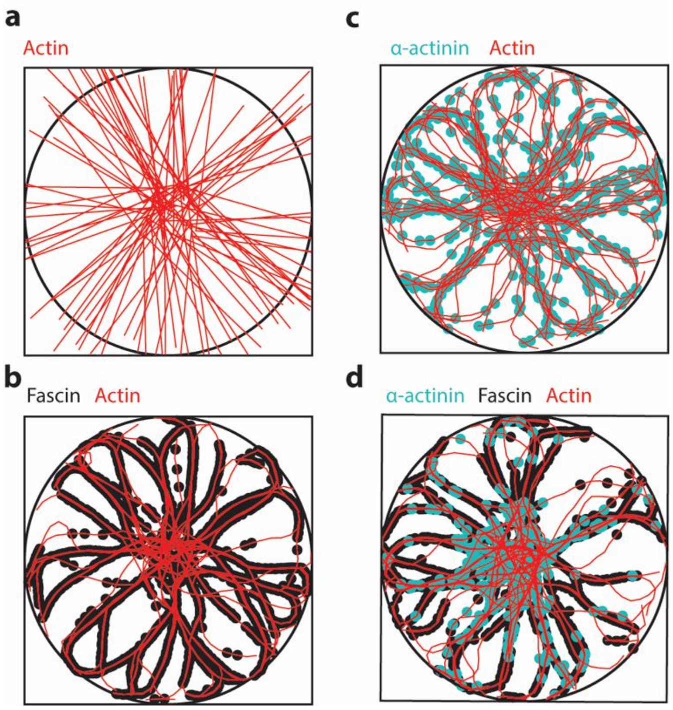

Supplementary Figure 10. Simulated fascin and α-actinin form distinct structures when simulated alone or together on asters. a, A representative snapshot of the initial condition of one simulation. Actin is shown in red and the boundary of the containing circle is shown as a black outline. **b,** A representative snapshot from a simulation of fascin alone with actin after 100 seconds of simulation. Fascin is represented as black dots. **c,** A representative snapshot from a simulation of α-actinin alone with actin after 100 seconds of simulation. α-actinin is represented as cyan dots. **d,** A representative structure after 100 seconds of both crosslinkers simulated together with actin.

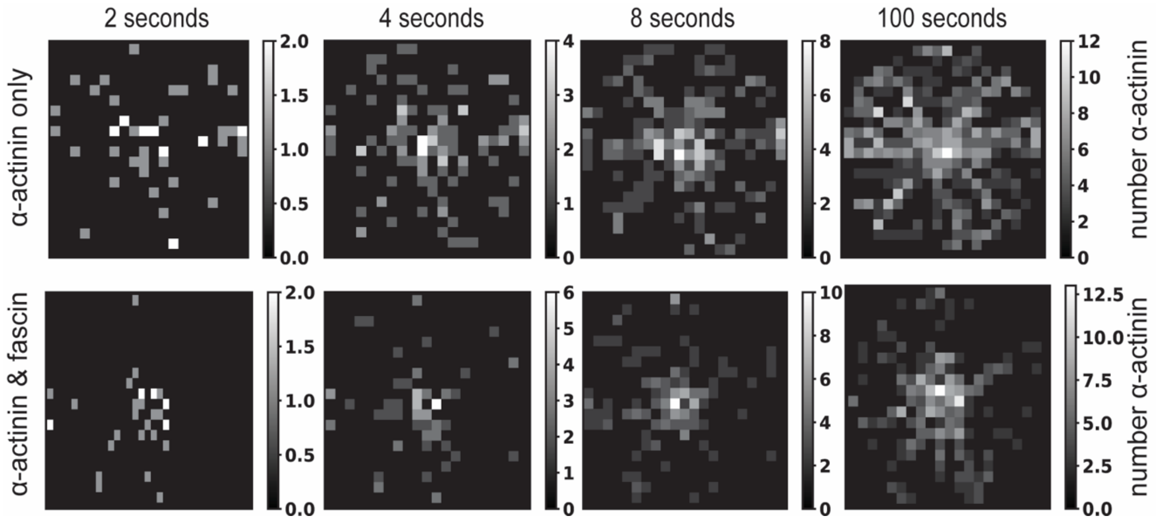

Supplementary Figure 11. α-actinin has a propensity for binding in the middle of simulated asters. Time lapse histograms of the number of α-actinin bound in a simulation. The top row is a simulation with α-actinin alone while the bottom row is taken from a combined simulation. Note that in both cases the center of the frame is preferentially occupied first.

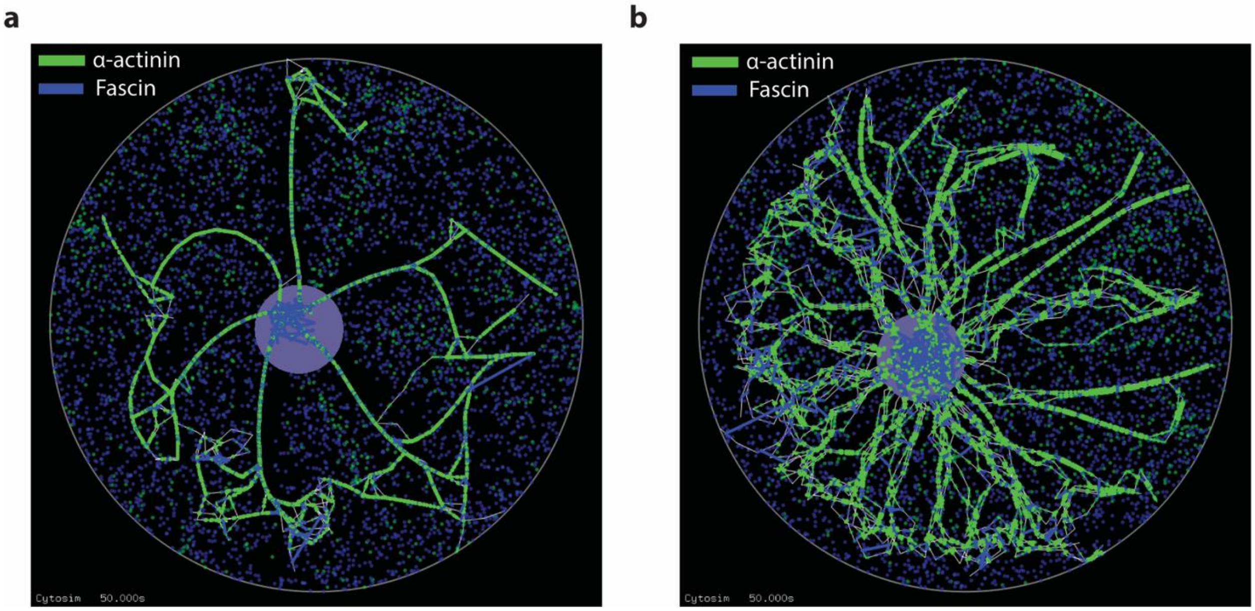

Supplementary Figure 12. Sorting of α-actinin and fascin to distinct regions can be seen by using the Cytosim package. Structures of asters in Cytosim crosslinked by our representations of α-actinin (blue) and fascin (green) after 50 s of simulation. **a,** Structure resultant from including fascin's description in the Cytosim fork feature (see Methods). **b,** Structure resultant from parametrizing fascin not with the fork feature but merely as a crosslink.

Supplemental Information

Supplementary Tables

Supplementary Table 1. List of primers for Gibson assembly

Primer	Sequence
pGEX-6P-1-FWD	TGACTGACTGACGATCTGCCTC
pGEX-6P-1-REV	GGGCCCCTGGAACAGAACTTC
Fascin-FWD	TCGGATCTGGAAGTTCTGTTCCAGGGGCC CACCGCCAACGGCACAGCC
Fascin-REV	AAACGCGCGAGGCAGATCGTCAGTCAGT CACTAGTACTCCCAGAGCGAGGCG
pGEX-Fascin-1-FWD	ACCGCCAACGGCACAGCC
pGEX-Fascin1-REV	TCACGCTCGTCGTTTGGTATGGC
pGEX-Fascin2-FWD	CACCACGATGCCTGCAGC
pGEX-Fascin2-REV	GGGCCCCTGGAACAGAAC
sfGFP-FWD	TCGGATCTGGAAGTTCTGTTCCAGGGGCC CTCCAAGGGTGAAGAGCTATTTACTGG
sfGFP-REV	CTGCACCGCCTCGGCTGTGCCGTTGGCGG TGCCGCTGCTGCCCTTATAAAGC

Supplementary Table 2. Simulation parameters of fascin and α-actinin.

Crosslinkers properties	Fascin	α-Actinin
resting length (μm)	0.1	0.5
on rate (s^{-1})	20	0.2
off rate (s^{-1})	0.5	0.05
k_{xl}^{align} ($pN \cdot \mu m$)	0.333	0
κ ($\frac{pN}{\mu m}$)	1	0.1

Supplementary Table 3. Parameters of the simulation box and actin.

**Simulation parameters**	
X range (μm)	100
Y range (μm)	100
r_c (μm)	15
k_c ($\frac{pN}{\mu m}$)	20
link length (μm)	1
polymer bending modulus ($pN \cdot \mu m^2$)	0.068
number of polymers	50
beads per polymer	35
time step (s)	2×10^{-5}
end time (s)	100

Supplementary References

1. Schneider CA, Rasband WS, Eliceiri KW. NIH Image to ImageJ: 25 years of image analysis. *Nature Methods* 2012, **9**(7)**:** 671-675.

2. Schindelin J, Arganda-Carreras I, Frise E, Kaynig V, Longair M, Pietzsch T, *et al.* Fiji: an open-source platform for biological-image analysis. *Nature Methods* 2012, **9**(7)**:** 676-682.

3. Rizk A, Paul G, Incardona P, Bugarski M, Mansouri M, Niemann A, *et al.* Segmentation and quantification of subcellular structures in fluorescence microscopy images using Squassh. *Nature Protocols* 2014, **9**(3)**:** 586-596.

4. Xu T, Vavylonis D, Huang X. 3D actin network centerline extraction with multiple active contours. *Medical image analysis* 2014, **18**(2)**:** 272-284.

5. Xu T, Vavylonis D, Tsai F-C, Koenderink GH, Nie W, Yusuf E, *et al.* SOAX: A software for quantification of 3D biopolymer networks. *Scientific Reports* 2015, **5**(1)**:** 9081.

6. Thevenaz P, Ruttimann UE, Unser M. A pyramid approach to subpixel registration based on intensity. *IEEE transactions on image processing* 1998, **7**(1)**:** 27-41.

7. Pettersen EF, Goddard TD, Huang CC, Couch GS, Greenblatt DM, Meng EC*, et al.* UCSF Chimera—A visualization system for exploratory research and analysis. *Journal of Computational Chemistry* 2004, **25**(13)**:** 1605-1612.
