# Supplementary material for: Actin crosslinker competition and sorting drive emergent GUV size-dependent actin network architecture
Source: Commun Biol. 2021 Sep 28;4:1136. doi: 10.1038/s42003-021-02653-6 (PMC8478941; doi:10.1038/s42003-021-02653-6)
Supplement: Supplementary file 3 — Reporting Summary [file 42003_2021_2653_MOESM3_ESM.pdf]

nature research

Corresponding author(s): DBPR, COMMSBIO-21-1143-T

Last updated by author(s): Aug 23, 2021

Reporting Summary

Nature Research wishes to improve the reproducibility of the work that we publish. This form provides structure for consistency and transparency in reporting. For further information on Nature Research policies, see our Editorial Policies and the Editorial Policy Checklist.

Statistics

For all statistical analyses, confirm that the following items are present in the figure legend, table legend, main text, or Methods section.

n/a	Confirmed	
☐	☒	The exact sample size (n) for each experimental group/condition, given as a discrete number and unit of measurement
☐	☒	A statement on whether measurements were taken from distinct samples or whether the same sample was measured repeatedly
☐	☒	The statistical test(s) used AND whether they are one- or two-sided<br>*Only common tests should be described solely by name; describe more complex techniques in the Methods section.*
☐	☒	A description of all covariates tested
☐	☒	A description of any assumptions or corrections, such as tests of normality and adjustment for multiple comparisons
☐	☒	A full description of the statistical parameters including central tendency (e.g. means) or other basic estimates (e.g. regression coefficient) AND variation (e.g. standard deviation) or associated estimates of uncertainty (e.g. confidence intervals)
☐	☒	For null hypothesis testing, the test statistic (e.g. F, t, r) with confidence intervals, effect sizes, degrees of freedom and P value noted<br>*Give P values as exact values whenever suitable.*
☒	☐	For Bayesian analysis, information on the choice of priors and Markov chain Monte Carlo settings
☒	☐	For hierarchical and complex designs, identification of the appropriate level for tests and full reporting of outcomes
☒	☐	Estimates of effect sizes (e.g. Cohen's d, Pearson's r), indicating how they were calculated

Our web collection on statistics for biologists contains articles on many of the points above.

Software and code

Policy information about availability of computer code

Data collection	We used our AFINES simulation package. It employs a coarse-grained representation of components to simulate cytoskeletal dynamics. We also used Cytosim simulation package to verify our model.
Data analysis	Encapsulated actin structures were mostly analyzed using ImageJ/Fiji, complemented with the plugin Squassh from the MOSAIC ToolSuite update site, in combination with SOAX source code. MATLAB (R2019b) routines were developed for illustration and characterization of encapsulated actin networks in UCSF Chimera (version 1.13.1) and Origin (2019b) respectively. Data were statistically analyzed in Excel worksheets.

For manuscripts utilizing custom algorithms or software that are central to the research but not yet described in published literature, software must be made available to editors and reviewers. We strongly encourage code deposition in a community repository (e.g. GitHub). See the Nature Research guidelines for submitting code & software for further information.

Data

Policy information about availability of data

All manuscripts must include a data availability statement. This statement should provide the following information, where applicable:

- Accession codes, unique identifiers, or web links for publicly available datasets
- A list of figures that have associated raw data
- A description of any restrictions on data availability

The experimental datasets obtained during this study are available in the "figshare" repository, https://figshare.com/articles/journal_contribution/Actin_crosslinker_competition_and_sorting_drive_emergent_GUV_size-dependent_actin_network_architecture/16337442.
The updated version of AFINES simulation package is available at https://github.com/dinner-group/AFINES/tree/sorting_guv/crosslinker_sorting_in_GUVs.
The data that support the findings of this study are available from the corresponding author upon reasonable request.

Field-specific reporting

Please select the one below that is the best fit for your research. If you are not sure, read the appropriate sections before making your selection.

☒ Life sciences ☐ Behavioural & social sciences ☐ Ecological, evolutionary & environmental sciences

For a reference copy of the document with all sections, see nature.com/documents/nr-reporting-summary-flat.pdf

Life sciences study design

All studies must disclose on these points even when the disclosure is negative.

Sample size	Number of GUVs per independent experiment were determined by direct visualization of GUVs in ImageJ/Fiji.
Data exclusions	GUVs encapsulating fluorescent actin monomers with no sign of bundling activity were occasionally found in each population. These GUVs were not taken into account for probability distribution and percentage measurements.
Replication	3 independent experiments were conducted at each condition to verify reproducibility. All attempts at replication were successful.
Randomization	Randomization of data was not required as we measured physical values, i.e. GUV size, bundle length, bundle curvature, actin and crosslinker density, and bundle persistence length.
Blinding	Experimental design was not blinded.

Reporting for specific materials, systems and methods

We require information from authors about some types of materials, experimental systems and methods used in many studies. Here, indicate whether each material, system or method listed is relevant to your study. If you are not sure if a list item applies to your research, read the appropriate section before selecting a response.

Materials & experimental systems

n/a	Involved in the study	
☒	☐	Antibodies
☒	☐	Eukaryotic cell lines
☒	☐	Palaeontology and archaeology
☒	☐	Animals and other organisms
☒	☐	Human research participants
☒	☐	Clinical data
☒	☐	Dual use research of concern

Methods

n/a	Involved in the study	
☒	☐	ChIP-seq
☒	☐	Flow cytometry
☒	☐	MRI-based neuroimaging
